# Long Non-coding RNA DLEU1 Promotes Proliferation and Invasion by Interacting With miR-381 and Enhancing HOXA13 Expression in Cervical Cancer

**DOI:** 10.3389/fgene.2018.00629

**Published:** 2018-12-07

**Authors:** Chang Liu, Xing Tian, Jing Zhang, Lifeng Jiang

**Affiliations:** ^1^Intensive Care Unit, Cancer Hospital Affiliated to Zhengzhou University, Zhengzhou, China; ^2^Department of Nephrology, The First Affiliated Hospital of Zhengzhou University, Zhengzhou, China; ^3^Department Gynecologic Tumor, Cancer Hospital Affiliated to Zhengzhou University, Zhengzhou, China; ^4^Department of Chinese and Western Medicine, Cancer Hospital Affiliated to Zhengzhou University, Zhengzhou, China

**Keywords:** DLEU1, long non-coding RNA, cervical cancer, miR-381, HOXA13

## Abstract

Although growing evidence has demonstrated that the long non-coding RNA DLEU1 is involved in the progression of various cancers, its functional role and underlying mechanisms have not been explored in cervical cancer (CC). In this study, we found that DLEU1 was up-regulated in both CC tissues and CC cell lines, and overexpression of DLEU1 was significantly correlated with shorter patient survival. Knockdown of DLEU1 suppressed CC cell proliferation and invasion, whereas overexpression of DLEU1 promoted the proliferation and invasion of CC cells. Bioinformatics analysis was used to elucidate the potential correlation between DLEU1 and miR-381. Moreover, qRT-PCR analysis, luciferase reporter assay and RNA immunoprecipitation assay confirmed that DLEU1 inhibited the expression of miR-381, and revealed a direct interaction between DLEU1 and miR-381. In addition, we demonstrated that miR-381 directly targeted HOXA13 in CC cells. The restoration of HOXA13 expression reversed DLEU1 knockdown or miR-381 overexpression-mediated suppression of cell proliferation and invasion. These results suggested that DLEU1 can promote CC cell proliferation and invasion via the miR-381/HOXA13 axis.

## Introduction

Cervical cancer (CC) is the second most common cause of death among women with various cancers, with over 500,000 new patients diagnosed and approximately 280,000 deaths each year (Torre et al., 2015). Although CC is curable if diagnosed at an early stage, many still present with an advanced or metastatic disease and have a worse prognosis. Metastasis is responsible for as much as 90% of cancer-induced mortality (Chaffer and Weinberg, 2011). Consequently, a better understanding of the molecular mechanism in metastatic CC is essential for the development of effective therapeutic strategies against CC.

MicroRNAs (miRNAs) and long non-coding RNAs (lncRNAs) have been shown to have crucial roles in carcinogenesis, metastasis and drug resistance (Han Li and Chen, 2015). MiRNAs post-transcriptionally regulate the expression of oncogenes and tumor suppressor genes, thus modulating the biological behaviors of tumor cells (Fabian et al., 2010). LncRNAs are known to function as scaffolds or guides to regulate interactions between protein and genes, and as decoys to bind proteins or miRNAs to modulate the expression of their target genes (Sanchez Calle et al., 2018). Importantly, lncRNAs act as competing endogenous RNAs (ceRNAs) and participate in a miRNA-dependent crosstalk by competitively binding miRNAs, representing a critical mechanism of tumor development and metastasis (de Giorgio et al., 2013; Gao et al., 2017).

LncRNA DLEU1, located on chromosome 13q14.3, has been reported to be dysregulated in chronic lymphocytic leukemia, multiple myeloma, breast cancer, gastric cancer and ovarian cancer (Garding et al., 2013; Dowd et al., 2015; Wu et al., 2015; Wang et al., 2017; Li et al., 2018). High expression of DLEU1 was associated with poor prognosis of gastric cancer and contributes to cell proliferation (Li et al., 2018). DLEU1 was also shown to promote ovarian cancer cell proliferation and invasion by interacting with miR-490-3p (Wang et al., 2017). However, little is known about the role and the upstream regulatory mechanism of DLEU1 in CC.

In our current study, we found that DLEU1 was highly expressed in CC tissues and CC cell lines. Moreover, silencing DLEU1 expression obviously inhibited the proliferative and invasive ability of CC cells. Mechanistic analysis demonstrated that DLEU1 served as a ceRNA by sponging miR-381 and upregulating HOXA13 expression, thus promoting CC cell proliferation and invasion. The DLEU1/miR-381/HOXA13 axis might be a promising therapeutic target for CC.

## Materials and Methods

### Cell Cultures and Transfection

Human CC cell lines (SiHa cells and HeLa cells) and human normal cervical cells (Academia Sinica Cell Bank, Shanghai, China) were conserved in DMEM/F12 (GIBCO-BRL) mediums with ten percent of fetal bovine serum (FBS) under a wettish condition at 37°C with 5% of CO_2_. Two different DLEU1 siRNAs (Genepharma, Shanghai, China) were used for knockdown of DLEU1 expression. Scrambled siRNA was used as a negative control (Genepharma, Shanghai, China). miR-381 mimic, control mimic, miR-381 inhibitor and control inhibitor were purchased from Genepharma (Shanghai, China). The DLEU1 expression plasmid pcDNA3.1-DLEU1 and the HOXA13 expression plasmid pcDNA3.1-HOXA13 were constructed by Genepharma (Shanghai, China). The empty vector pcDNA3.1 was used as negative control. Lipofectamine 2000 (Invitrogen, Carlsbad, CA, United States) was used for cell transfection following the guidance of the manufacturer’s instructions.

### Quantitative Real-Time PCR (qRT-PCR)

The expression level of DLEU1 was detected by real-time RT-PCR. In brief, total RNA from CC cells was extracted using TRIzol reagent (Invitrogen, Carlsbad, CA, United States) according to the manufacturer’s instructions. We reversely transcribed 1 μg of RNA into cDNA in virtue of a Reverse Transcription Kit (Takara, Dalian, China). Real-time PCR analyses were performed using SYBR-Green-quantitative real-time PCR Master Mix kit (Toyobo Co., Osaka, Japan). The mirVanaTM qRT-PCR microRNA Detection Kit (Ambion Inc., Austin, TX, United States) was used for miR-381 detection according to the manufacturer’s instructions. A specific stem-loop RT primer was used for miR-381 detection. The primer sequences used have been reported (Lee et al., 2017). MiR-381 was normalized to U6. DLEU1 expression data were normalized to GAPDH.

### Western Blot Analysis

The entire protein lysates were subjected to 10% sodium dodecyl sulfate-polyacrylamide gel electrophoresis (SDS-PAGE) and then transferred to a PVDF membrane (Millipore, Bedford, MA, United States). Then the above PVDF membranes were incubated with the corresponding primary antibody HOXA13 (1:2000, Abcam, Cambridge, United Kingdom) and GAPDH (1:1000, Santa Cruz Biotech, Santa Cruz, CA, United States) overnight, followed by incubation with HRP-conjugated secondary antibodies (Santa Cruz, CA, United States). Protein bands were detected using an ECL western blotting kit (Amersham Biosciences, Buckinghamshire, United Kingdom). GAPDH was used as the loading control.

### CCK-8 Assay

Cervical cancer cells (5 × 10^3^ cells per well in 96-well plates) were transfected as described above. Cell proliferation was measured 72 h after transfection using a CCK-8 (Beyotime Institute of Biotechnology, Jiangsu, China) according to the manufacturer’s instructions. The absorbance was measured at 450 nm by a microplate reader (Bio-Rad, Hercules, CA, United States).

### *In vitro* Invasion Assay

The invasive ability of the cells was measured using transwell chambers (Corning, New York, United States), as described previously (Dong P. et al., 2017). In brief, cells (5 × 10^4^) suspended in serum-free medium were transferred to the upper chamber. The medium containing 10% FBS was added as chemokine in the lower chamber. After 24 h, the invaded cells on the membrane lower surface were fixed with 75% methanol, and stained with crystal violet. Evaluation of invasive capacity was performed by counting invading cells under a microscope, and five random fields of view were analyzed for each chamber. All experiments were performed in triplicate.

### Luciferase Reporter Assay

The wild-type DLEU1 (DLEU1-WT), mutant DLEU1 (DLEU1-MUT), wild-type HOXA13 3′-UTR (HOXA13-WT), and mutant HOXA13 3′-UTR (HOXA13-MUT) were synthesized and cloned into pMIR-GLO^TM^ Luciferase vectors (Promega, Madison, WI, United States). For the luciferase reporter assay, CC cells were co-transfected with the above luciferase reporter vectors containing DLEU1 (WT or MUT) or HOXA13 3′-UTR (WT or MUT) and miR-381 mimic, miR-381 inhibitor or their respective controls using Lipofectamine 2000 (Invitrogen). Luciferase activity was measured after 48 h. The relative luciferase activity was measured with the Dual-Luciferase Reporter Assay System (Promega, China). Firefly luciferase activity was normalized to that of Renilla luciferase.

### RNA Immunoprecipitation Assay (RIP)

To verify the interaction between DLEU1 and miR-381, RNA immunoprecipitation assay was conducted using the Magna RIP^TM^ RNA-Binding Protein Immunoprecipitation Kit (Millipore). Briefly, CC cells at 80% confluency were harvested and lysed in complete RIP lysis buffer. Then, the whole cell extract was co-immunoprecipitated with RIP buffer containing magnetic beads conjugated with anti-Argonaute2 (Ago2) antibody (Millipore, Bedford, MA, United States) or normal mouse IgG (Millipore) as a negative control. Samples were digested with proteinase K, and RNAs were isolated from the immunoprecipitation products were subjected to qRT-PCR analysis of DLEU1 and miR-381 expression.

### Statistical Analysis

All statistical analyses were performed using SPSS 17.0 statistical software (IBM, Armonk, NY, United States). Data are presented as the mean ± standard deviation (SD) from at least three experiments. The significant differences were analyzed using Student’s *t*-test or one-way ANOVA. *P*-values were based on the two-sided statistical analysis, and a *P*-value < 0.05 from a two-tailed test was considered significant.

### Data Availability

All data generated during this study are available from the corresponding author on reasonable request.

## Results

### DLEU1 Is Upregulated in CC Tissues and CC Cell Lines and Significantly Correlated With Shorter Patient Survival

**FIGURE 1:**
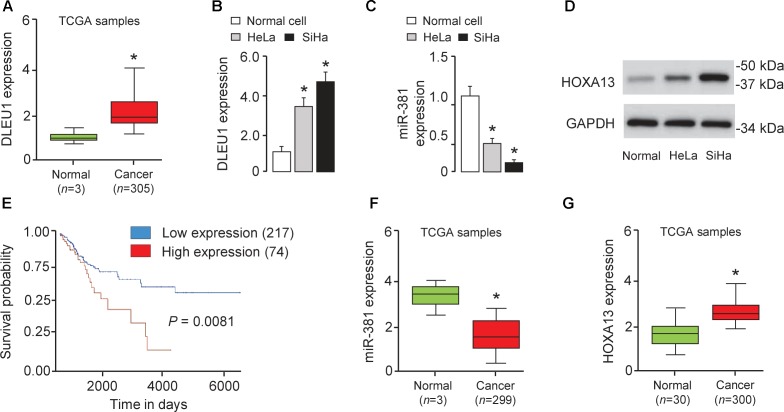
Overexpression of DLEU1 is associated with poor survival in patients with CC. **(A)** Expression of DLEU1 in CC samples (*n* = 305) and normal cervical tissues (*n* = 3). The Cancer Genome Atlas (TCGA) datasets were retrieved in the UALCAN web server. **(B–D)** The differential expression of DLEU1 **(B)**, miR-381 **(C)** and HOXA13 **(D)** in CC cell lines and normal cervical cell were examined as indicated. **(E)** Kaplan-Meier survival analysis of CC patients with DLEU1 expression and outcome data. Expression of miR-361 **(F)** and HOAX13 **(G)** in CC tissues and normal tissues from TCGA datasets. ^∗^*p* < 0.05.

To investigate the role of lncRNA in CRC metastasis, we first analyzed the expression of DLEU1 in CC tissues and normal cervical tissues using the TCGA database (Chandrashekar et al., 2017). We found that the level of DLEU1 was markedly increased in CC tissues in comparison with that in the normal samples (Figure 1A). Subsequently, we investigated the expression of DLEU1 in CC cell lines (SiHa cells and HeLa cells) and human normal cervical cells. Our qRT-PCR analysis suggested that the level of DLEU1 was significantly higher in CC cells compared with the normal cervical cells (Figure 1B), indicating that DLEU1 might have an oncogenic role in CC progression. To determine the association between DLEU1 expression and patient survival, we examined the expression of DLEU1 in TCGA dataset via the UALCAN web server. For the cohort of 291 patients, overexpression of DLEU1 was associated with poor survival (Figure 1E). The difference in survival is significant (*P* = 0.008). These data indicate that DLEU1 expression may be an important prognostic factor for patients with CC.

### Overexpression of DLEU1 Promotes CC Cell Proliferation and Invasion

**FIGURE 2:**
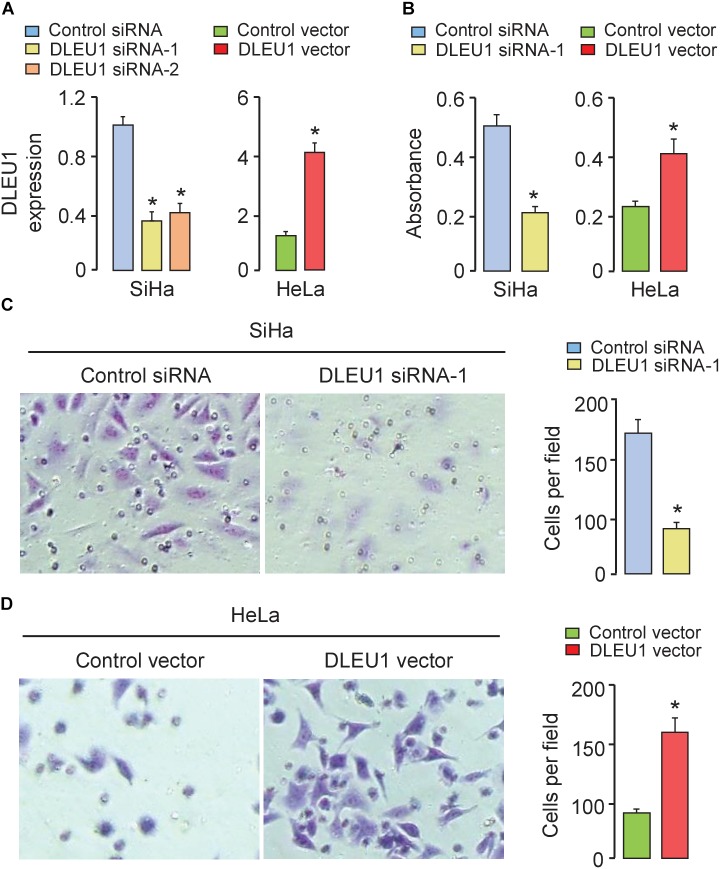
Silencing of DLEU1 inhibited, whereas overexpression of DLEU1 promoted proliferation and invasion of CC cells. **(A)** qRT-PCR analysis of DLEU1 expression in SiHa cells transfected with DLEU1 siRNA-1, DLEU1-siRNA-2 or control siRNA, and in HeLa cells transfected with pcDNA3.1-DLEU1 or empty vector pcDNA3.1. **(B)** CCK-8 assay was used to measure cell proliferation in SiHa cells transfected with DLEU1 siRNA-1 or control siRNA, and in HeLa cells transfected with pcDNA3.1-DLEU1 or empty vector pcDNA3.1. **(C,D)** Transwell invasion assay was performed to assess invasiveness in SiHa cells transfected with DLEU1 siRNA-1 or control siRNA **(C)**, and in HeLa cells transfected with pcDNA3.1-DLEU1 or empty vector pcDNA3.1 **(D)**. ^∗^*p* < 0.05.

To test the biological function of DLEU1 in CC cells, two different DLEU1-specific siRNAs were used to silence DLEU1 expression in SiHa cells, which exhibits the high level of DLEU1. The qRT-PCR analysis confirmed down-regulation of DLEU1 levels in SiHa cells. Both siRNA-1 and siRNA-2 resulted in a significant down-regulation of DLEU1 expression (Figure 2A). The transfection with siRNA-1 was more effective than the transfection with siRNA-2 in terms of downregulating the DLEU1 level. We therefore chose to use DLEU1-siRNA-1 for all subsequent experiments.

Furthermore, HeLa cells with low expression of DLEU1 were selected in the following experiments. We stably overexpressed DLEU1 and found that DLEU1 level was significantly elevated after transfecting with pcDNA3.1-DLEU1 in HeLa cells (Figure 2A). To assess the influence of DLEU1 on cell proliferation and invasion, either loss-of-function or gain-of-function assays were performed. Results of CCK-8 assay and invasion assay showed that knockdown of DLEU1 significantly weakened the proliferative and invasive ability of SiHa cells (Figures 2B,C). However, overexpression of DLEU1 increased the proliferation and invasion of HeLa cells, as measured using the CCK-8 assay and invasion assay (Figures 2B,D). These data indicated that overexpression of DLEU1 promoted CC cell growth and invasion.

### DLEU1 Acts as a ceRNA by Sponging miR-381 in CC Cells

**FIGURE 3:**
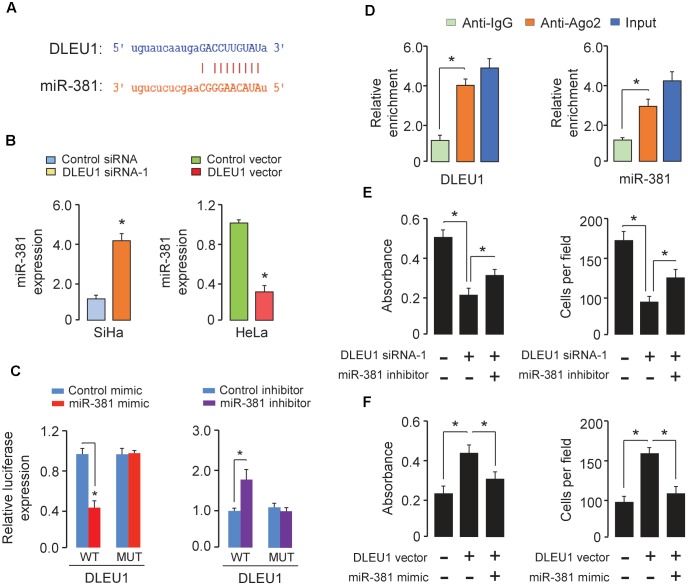
DLEU1 acts as a ceRNA by sponging miR-381. **(A)** The predicted binding site of miR-381 to the DLEU1 sequence was shown. **(B)** qRT-PCR analysis of miR-381 in SiHa cells transfected with DLEU1 siRNA-1 or control siRNA, and in HeLa cells transfected with pcDNA3.1-DLEU1 or empty vector pcDNA3.1. **(C)** The relative luciferase activity in SiHa cells cotransfected with luciferase reporter vectors containing wild-type (WT) DLEU1 or mutant (MUT) DLEU1 and control mimic or miR-381 mimic, and in HeLa cells cotransfected with luciferase reporter vectors containing wild-type DLEU1 or mutated DLEU1 and miR-381 inhibitor or control inhibitor. **(D)** RIP assay was performed in SiHa cells. DLEU1 and miR-381 expression was detected using qRT-PCR. **(E)** Cell proliferation (left panel) and invasion (right panel) assessed in SiHa cells transfected with control siRNA + control inhibitor, DLEU1 siRNA-1 + control inhibitor, or DLEU1 siRNA-1 + miR-381 inhibitor. **(F)** Cell proliferation (left panel) and invasion (right panel) assessed in HeLa cells transfected with control vector + contro1 mimic, DLEU1 vector + contro1 mimic, or DLEU1 vector+ miR-381 mimic. ^∗^*p* < 0.05.

To investigate whether DLEU1 functions as a molecular sponge of miRNA to liberate mRNA transcript targeted by miRNA, thereby contributing to CC progression, we used the public prediction algorithm StarBase V2.0 (Li et al., 2014) and identified miR-381 with complementary sequences to the DLEU1 transcript (Figure 3A). Some ceRNAs will degrade their miRNA binding partners (Liu et al., 2017).

To study the clinical relevance of miR-381 to human CC, we examined miR-381 expression in normal cells and CC cell lines. The level of miR-381 was significantly downregulated in SiHa and HeLa cells than the normal cells (Figure 1C). Using the BioExpress database^1^, we analyzed the TCGA data to evaluate miR-381 expression in human CC tissues and normal tissues. We found that miR-381 expression was markedly downregulated in CC tissues compared with normal tissues (Figure 1F). These results suggested a negative correlation between DLEU1 and miR-381 expression in CC (Figure 1A).

To examine whether DLEU1 has an impact on miR-381 expression, qRT-PCR was used to investigate the effect of DLEU1 knockdown or overexpression on miR-381 expression in CC cells. MiR-381 expression was up-regulated in SiHa cells with DLEU1 knockdown (Figure 3B). In contrast, overexpression of DLEU1 reduced the expression of miR-381 in HeLa cells (Figure 3B).

To further whether DLEU1 directly interacts with miR-381, we constructed luciferase reporter vectors containing wild-type or mutated DLEU1 and performed luciferase reporter assay. As shown in Figure 3C, ectopic expression of miR-381 resulted in a significant reduction in luciferase activity of wild-type DLEU1 in SiHa cells (Figure 3C), but had no evident inhibitory effect on mutant DLEU1. In addition, the transfection with miR-381 inhibitor led to a notable increase in the luciferase activity of wild-type DLEU1 in HeLa cells, while miR-381 did not affect the luciferase activity of mutated DLEU1 (Figure 3C).

In order to further verify the direct binding between miR-381 and DLEU1 at endogenous levels, RIP assay was performed to pull down endogenous miRNAs associated with DLEU1 in SiHa cells using the antibody against Ago2. We found that DLEU1 and miR-381 were specifically enriched in Ago2 pellets of SiHa cell extracts relative to the IgG control group (Figure 3D).

To determine whether DLEU1 exerted its function through miR-381 in CC cells, we performed the rescue experiments by transfecting DLEU1 siRNA in combination with miR-381 inhibitor into SiHa cells, or by transfecting DLEU1 vector in combination with miR-381 mimic into HeLa cells. The knockdown of DLEU1 significantly impeded cell proliferation and invasion of SiHa cells, while the inhibition of miR-381 significantly abrogated these effects (Figure 3E). Conversely, the induction in proliferative capacity and invasion ability caused by overexpressing DLEU1 could be largely reversed by the restoration of miR-381 in HeLa cells (Figure 3F). Taken together, our results indicated that DLEU1 could serve as a ceRNA by binding miR-381 in CC cells.

### miR-381 Targeted HOXA13 in CC Cells

**FIGURE 4:**
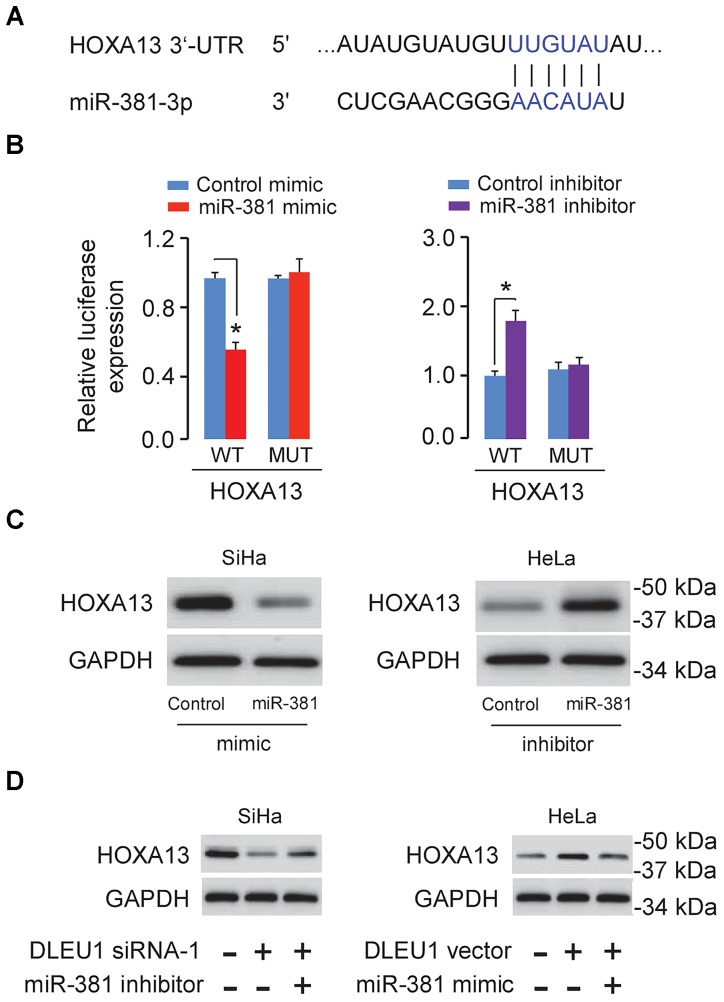
DLEU1 indirectly induced HOXA13 expression via inhibiting miR-381 expression. **(A)** Predicted miR-381 binding site in the 3′-UTR of HOXA13. **(B)** The relative luciferase activities of reporter vectors in SiHa cells (left panel) cotransfected with luciferase reporter vectors containing wild-type (WT) HOXA13 or mutated (MUT) HOXA13 and control mimic or miR-381 mimic, and in HeLa cells (right panel) cotransfected with luciferase reporter vectors containing wild-type HOXA13 or mutated HOXA13 and miR-381 inhibitor or control inhibitor. **(C)** The protein level of HOXA13 in SiHa cells transfected with miR-381 mimic or control mimic, and in HeLa cells transfected with miR-381 inhibitor or control inhibitor. **(D)** The protein expression of HOXA13 in SiHa cells transfected with contro1 siRNA + control inhibitor, DLEU1 siRNA-1 + control inhibitor, or DLEU1 siRNA-1 + miR-381 inhibitor, and in HeLa cells transfected with contro1 vector pcDNA3.1 + control mimic, pcDNA3.1-DLEU1 + control mimic, or pcDNA3.1-DLEU1 + miR-381 mimic. ^∗^*p* < 0.05.

To further explore the potential targets of miR-381 in CC cells, we performed the bioinformatic-based target prediction analysis using TargetScan^2^. Among the potential target genes, HOXA13 was predicted to contain the binding sequence of miR-381 (Figure 4A), and its upregulation was shown to enhance cancer cell proliferation and invasion (He et al., 2017; Yu et al., 2018). Next, we explored the protein expression of HOXA13 in CC cells and normal cells. As demonstrated in Figure 1D, in comparison with that in normal cells, the expression of HOXA13 in CC cells was much higher, as measured using Western blot analysis, indicating that miR-381 directly targets HOXA13. We examined HOXA13 expression in TCGA CC datasets using the publicly available tool MethHC^3^, and found higher HOXA13 mRNA levels in CC tissues compared with normal tissues (Figure 1G), suggesting a positive correlation between DLEU1 in CC (Figure 1A).

Dual-luciferase reporter assay further showed that HOXA13 was the direct target of miR-381 (Figure 4B). As shown in Figure 4C, HOXA13 expression was decreased after transfecting with miR-381 mimic, but was increased after transfecting with miR-381 inhibitor.

### Restoration of HOXA13 Reversed the Effects of DLEU1 Knockdown or miR-381 Overexpression in CC Cells

Then, we conducted the rescue assays to further affirm the regulatory role of DLEU1 silencing and miR-381 inhibition on the protein expression of HOXA13 using Western blot analysis. The knockdown of DLEU1 reduced the protein level of HOXA13 in SiHa cells, while the transfection with miR-381 inhibitor apparently abolished this effect (Figure 4D). The induction of HOXA13 expression caused by DLEU1 overexpression could be largely suppressed by the transfection of miR-381 mimic in HeLa cells (Figure 4D), suggesting that DLEU1 liberated HOXA13 expression by competitively binding to miR-381.

**FIGURE 5:**
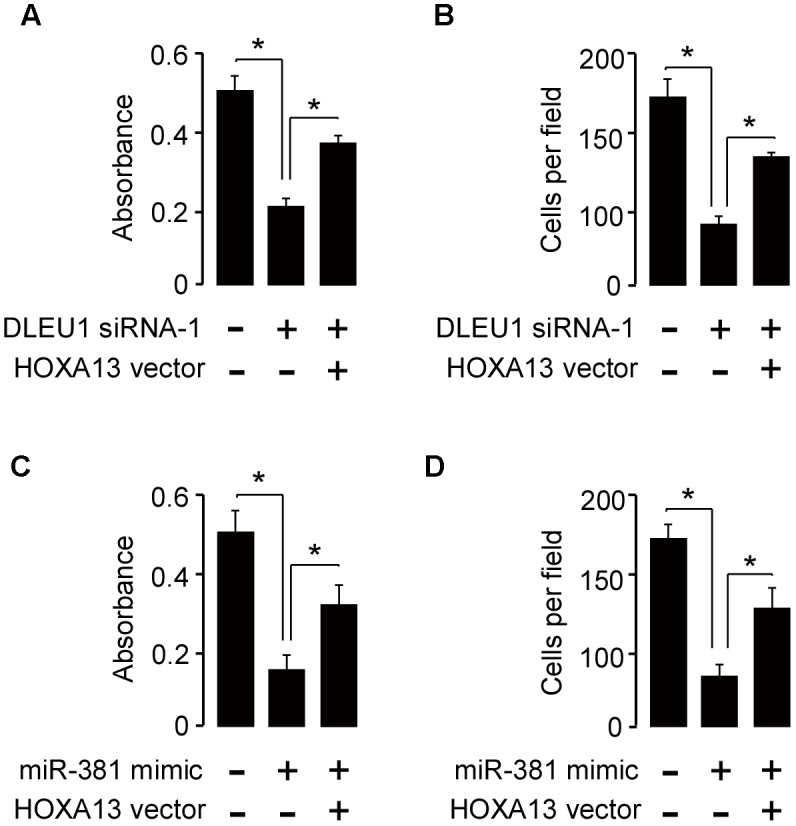
Restoration of HOXA13 reverses the inhibitory effects of DLEU1 knockdown or miR-381 overexpression on CC cell proliferation and invasion. **(A,B)** Cell proliferation **(A)** and invasion **(B)** assessed in SiHa cells transfected with control siRNA + contro1 vector pcDNA3.1, DLEU1 siRNA-1 + contro1 vector pcDNA3.1, or DLEU1 siRNA-1 + pcDNA3.1-HOXA13. **(C,D)** Cell proliferation **(C)** and invasion **(D)** assessed in SiHa cells transfected with control mimic + contro1 vector pcDNA3.1, miR-381 mimic + contro1 vector pcDNA3.1, or miR-381 mimic + pcDNA3.1-HOXA13. ^∗^*p* < 0.05.

Furthermore, CCK-8 and invasion assay showed that knockdown of DLEU1 or miR-381 overexpression significantly suppressed SiHa cell proliferation and invasion, while forced HOXA13 expression could partially rescue the anti-proliferation and anti-invasion effects mediated by the knockdown of DLEU1 or miR-381 overexpression (Figure 5). These data revealed that DLEU1 might promote CC cell growth and invasion by upregulating HOXA13 via competitively interacting with miR-381.

## Discussion

Emerging studies have revealed that lncRNA plays an important role in cancer progression, and they are aberrantly expressed in a variety of tumors including CC (Dong J. et al., 2017). For instance, lncRNA NORAD (Huo et al., 2018), TPT1-AS1 (Jiang et al., 2018) and NEAT1 (Guo et al., 2018) could promote cell growth and metastasis in CC. HOXD-AS1 motivates doxorubicin resistance in CC (Hou et al., 2018). HOTAIR and CCHE1 were overexpressed in CC tissues, and elevated expression of HOTAIR and CCHE1 were negative prognostic factors in CC (Kim et al., 2015; Chen et al., 2017). Up to now, studies have shown that DLEU1 functioned as an oncogenic lncRNA in several tumors (Garding et al., 2013; Dowd et al., 2015; Wu et al., 2015; Wang et al., 2017; Li et al., 2018), but the contribution of DLEU1 to CC cell proliferation and invasion and the underlying mechanisms still remained to be elucidated. In this study, we found that DLEU1 level was significantly increased in the CC tissues and CC cell lines. By performing loss-of-function and gain-of-function assays, our results revealed the oncogenic role of DLEU1 in promoting cellular proliferation and invasiveness. Thus, our study provided a new insight into the molecular mechanism of DLEU1 in CC progression.

Although lncRNAs are involved in many pathological processes, the underlying molecular mechanisms remain largely unknown. Recently, a novel mechanism was proposed in which lncRNAs could serve as ceRNAs for miRNAs in cancer (Salmena et al., 2011). For example, DLEU1 functions as a ceRNA for miR-490-3p, thereby up-regulating the expression of CDK1, CCND1 and SMARCD1 and subsequently promoting the development and progression of CC (Wang et al., 2017). Here, we show that DLEU1 acts as an oncogene *in vitro* through binding miR-381. Our luciferase activity assays and RIP assays further confirmed that DLEU1 served as a molecular sponge for miR-381 to upregulate the expression of its target HOXA13, thus promoting CC pathogenesis.

Previously, miR-381 was reported to be dysregulated in many cancers, including gastric cancer (Zhang et al., 2017), oral squamous cell carcinoma (Yang et al., 2017), breast cancer (Xue et al., 2017), colorectal cancer (He et al., 2016) and ovarian cancer (Xia et al., 2016). Moreover, miR-381 acts as a tumor suppressor and was known to widely participate in tumor cell proliferation, invasion, metastasis and chemoresistance (He et al., 2016; Xia et al., 2016; Cao et al., 2017; Xue et al., 2017; Yang et al., 2017; Zhang et al., 2017; Huang et al., 2018). However, little was known about its function in CC. In the present study, we reported that miR-381 could significantly suppress CC cell growth and invasion. Furthermore, we showed the endogenous interaction between DLEU1 and miR-381 by RIP assays in CC cells, thus our results also indicated that the effects of miR-381 on proliferation and invasion are possibly due to its direct interaction with DLEU1.

HOXA13 has been reported to be upregulated and has been demonstrated to play an oncogenic role in tumorigenesis and progression in various tumors (Dong Y. et al., 2017 He et al., 2017). Although HOXA13 was highly expressed in CC tissues compared to normal cervical tissues (Saha et al., 2017), its role in the proliferation and invasion of CC cells has not been reported. Through the bioinformatic analysis, we predicted the potential miRNA binding site in HOXA13. Importantly, we found that miR-381 directly interacted with HOXA13 and downregulated its expression in CC cells. The ectopic HOXA13 expression could partially rescued the suppressive effects of miR-381 mimic or DLEU1 knockdown on cell proliferation and invasion. These experiments supported that HOXA13 acts as an oncogene in CC, and DLEU1 exerted its function via miR-381/HOXA13 axis in CC cells.

## Conclusion

Taken together, these results revealed that DLEU1 might facilitate the progression of CC partially through the miR-381/HOXA13 axis. The DLEU1/miR-381/HOXA13 axis should be considered as a potential therapeutic target against CC.

## Author Contributions

CL provided the direction. CL, XT, and JZ performed the experiments. CL wrote the manuscript. LJ made significant revisions to the manuscript. All authors read and approved the final manuscript.

## Conflict of Interest Statement

The authors declare that the research was conducted in the absence of any commercial or financial relationships that could be construed as a potential conflict of interest.

## Footnotes

1 https://hive.biochemistry.gwu.edu/bioxpress/about

2 http://www.targetscan.org

3 http://methhc.mbc.nctu.edu.tw/php/index.php
